# Organ-on-a-Chip Models—New Possibilities in Experimental Science and Disease Modeling

**DOI:** 10.3390/biom14121569

**Published:** 2024-12-09

**Authors:** Bartłomiej Wysoczański, Marcin Świątek, Anna Wójcik-Gładysz

**Affiliations:** 1 Department of Animal Physiology, The Kielanowski Institute of Animal Physiology and Nutrition, Polish Academy of Sciences, Instytucka 3, 05-110 Jablonna, Poland; 2 Department of Animal Breeding, Institute of Animal Sciences, Warsaw University of Life Sciences, Ciszewskiego 8, 02-786 Warsaw, Poland

**Keywords:** organ-on-a-chip, microfluidics, neurodegenerative diseases, neuroendocrinology, microphysiological system

## Abstract

‘Organ-on-a-chip’ technology is a promising and rapidly evolving model in biological research. This innovative microfluidic cell culture device was created using a microchip with continuously perfused chambers, populated by living cells arranged to replicate physiological processes at the tissue and organ levels. By consolidating multicellular structures, tissue–tissue interfaces, and physicochemical microenvironments, these microchips can replicate key organ functions. They also enable the high-resolution, real-time imaging and analysis of the biochemical, genetic, and metabolic activities of living cells in the functional tissue and organ contexts. This technology can accelerate research into tissue development, organ physiology and disease etiology, therapeutic approaches, and drug testing. It enables the replication of entire organ functions (e.g., liver-on-a-chip, hypothalamus–pituitary-on-a-chip) or the creation of disease models (e.g., amyotrophic lateral sclerosis-on-a-chip, Parkinson’s disease-on-a-chip) using specialized microchips and combining them into an integrated functional system. This technology allows for a significant reduction in the number of animals used in experiments, high reproducibility of results, and the possibility of simultaneous use of multiple cell types in a single model. However, its application requires specialized equipment, advanced expertise, and currently incurs high costs. Additionally, achieving the level of standardization needed for commercialization remains a challenge at this stage of development.

## 1. Introduction

The rapidly developing organ-on-a-chip (OOC) technology is a promising new tool for biological research. This approach uses a microfluidic cell culture device consisting of a microchip containing continuously perfused chambers with living cells structured to replicate physiological processes at both tissue and organ levels. OOC technology provides an innovative alternative to conventional in vitro cell culture models, including two-dimensional (2D) and three-dimensional (3D) cultures. While 2D models have long been utilized in research, they are unable to reproduce the complex microenvironment of organs or tissues [1]. In contrast, 3D models, which integrate biomaterials resembling the extracellular matrix, provide a closer approximation to physiological conditions [2]. Common examples of 3D models include spheroids and organoids, which simulate some of the architectural and functional characteristics of tissues and tumors [3,4]. However, they still fall short of fully mimicking the dynamic physiological processes occurring within living organisms [5]. This gap in replicating complex in vivo environments underscores the growing focus on developing advanced technologies such as OOC.

The complexity of the nervous system continues to fuel efforts toward advancements in therapies and drug development. However, this progress is often hindered by significant challenges, particularly the high costs involved [6]. As these expenses increase, there is a growing need for innovative methods that enhance research efficiency and optimize resource utilization. OOC technology has the potential to provide cost-effective and reliable solutions. The progress in OOC is largely attributable to advancements in microfluidics, a field that has been developing since the late twentieth century, contributing substantially to the creation and refinement of these microdevices [7].

The origin of OOC technology can be traced back more than two decades, with the development of the first microfluidic system for capillary electrophoresis manufactured using photolithography [8]. Since its invention in the 1950s, photolithography has been primarily used in semiconductor manufacturing and was not adapted for analyses using biological materials [9,10]. Consequently, another method, known as soft lithography, was developed to address this technological gap. In 1998, Xia and Whitesides [11] pioneered this technique, which diverged from traditional methods by employing an optically transparent polymer and replica molding. This microfabrication method utilized a silicon-based organic polymer, poly(dimethylsiloxane) (PDMS), as the primary material.

Since the invention of soft lithography, microfluidic technologies have become a highly desired tool in biological research. Their diverse applications include drug development, biomedical research, omics studies, and environmental testing [12]. The integration of several bioengineering disciplines within the OOC, including microfluidics, 3D cell culture, and tissue engineering, positions this technology as a promising approach for preclinical research [5]. This represents a significant advancement beyond traditional cell and tissue culture methods, particularly in neuroscience research, where OOC models can aid in studying neurodegenerative disorders and developing new therapies and drugs [13,14]. The implementation of OOC technology can reduce reliance on animal models, as samples from fewer animals can be used across multiple OOC systems, significantly decreasing the number of animals required for experimentation [15,16].

## 2. Organ-on-a-Chip Technology

**Figure 1:**
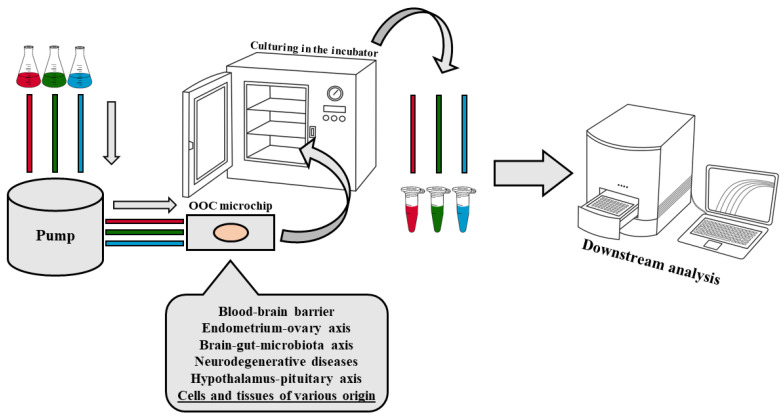
Schematic diagram of the basic OOC model design. The red, green, and blue colors shown in the figure represent the potential different media/test factors flowing through the microchip in any order. The design of the OOC allows for discretion in the number of media and/or test agents, as well as the order in which they are introduced into the OOC microenvironment.

OOC technology represents a significant advancement in the evolution of cell and tissue culture, while maintaining core principles of traditional culturing techniques [17]. These microfluidic devices simulate organ functions and disease states using organ-specific cells placed in parallel channels separated by a porous membrane (Figure 1). The system’s automated fluid flow and mechanical strain create physiologically relevant conditions, enabling the cells to mimic native organ behavior. These models allow for real-time data collection and traditional analyses, providing a reliable framework for studying drug effects and disease mechanisms [15,18]. When planning neurological studies using OOC models, three primary factors must be considered: defining the research model, selecting the research variable, and designing the OOC microchip. The selection of the research model is often based on the origin and availability of cell lines or tissue samples, including those derived from both animal and human cell lines [15,19]. A critical step is choosing the research factor that may influence the physiology of the designed model. The research factor can be physical (mechanical stress), chemical (drug candidate, peptides), or their combination [20,21,22]. Lastly, the entire OOC model is designed to provide optimal culture conditions and continuous monitoring capabilities, making it suitable for many applications and effective at recreating the physiological conditions of organs [14]. Additionally, the system includes apparatus for measuring specific parameters that reflect the culture’s response to the chosen experimental factor.

The initial step in designing an OOC model is selecting between recreating a single organ or combining multiple organs within the model. This aspect is crucial for the entire model, as it influences the entire model’s design, its anatomical accuracy, and the required devices for the chip. The existing literature provides evidence of successful attempts to mimic the significant systems responsible for neural regulation, such as the brain, blood–brain barrier (BBB), brain–gut–microbiota axis, and neurodegenerative disorders [23,24,25,26]. Integrating cells from different organs within a single chip allows for a more comprehensive approach to drug screening and toxicology, biomarker detection, cancer growth research, as well as disease modeling, such as Parkinson’s disease [27,28,29]. The wide selection of cell lines offers considerable flexibility when selecting the appropriate research materials. Compared to traditional cell cultures, OOC technology offers a more detailed and accurate representation of the pharmacokinetic and pharmacodynamic processes of the investigated compounds.

Another crucial aspect to consider in the design of OOC models is the selection of experimental agents. An important area of interest is drug screening, where various drug candidates are tested for their effects on a single or multiple organs replicated on a microchip [13,30]. This approach allows for detailed studies into the efficacy and toxicity of drug candidates in a controlled environment [31]. In addition to chemical testing, OOC models also allow researchers to examine the impact of various physical stimuli, including temperature fluctuations, UV irradiation, or mechanical stimulation [14,32,33]. By simulating these real-life conditions, OOC models provide valuable insights into how different factors affect organ systems, ultimately facilitating the development of more effective treatments and therapies.

The third aspect involves controlling the environmental parameters within OOC models to replicate the complex and dynamic conditions of a living organism. OOC microdevices offer precise control of the cellular microenvironment, including gas concentrations (oxygen and carbon dioxide), pH level, and medium flow rates. This level of control allows for comprehensive investigations of drug toxicity and efficacy, as well as the effects of various physical stimuli, including temperature fluctuations and mechanical pressure applied to the tissues under study [32,33,34]. Additionally, these microdevices can be integrated with precision monitoring sensors, which maintain an environment as close to physiological conditions as possible [35].

**Figure 2:**
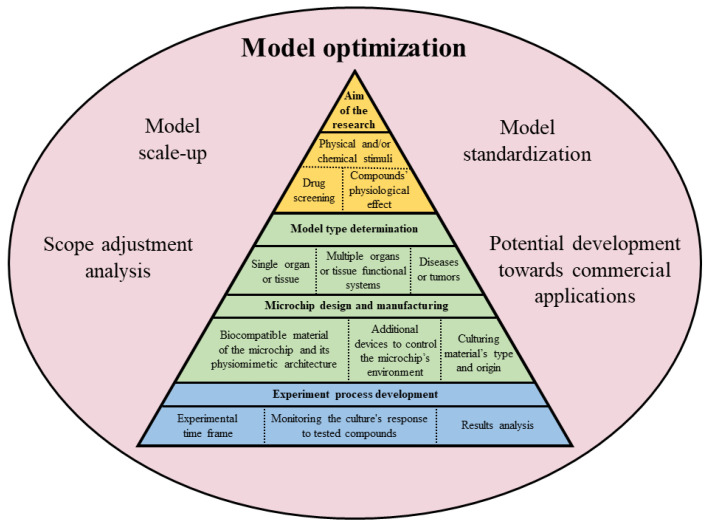
Development process for the experiments utilizing OOC as an alternative to animal models.

To summarize, designing OOC models requires addressing both biological and technical challenges to recreate accurate tissue system models. From a technical perspective, this involves replicating the complex architecture of target organs using design elements such as microfluidic channels, semi-permeable membranes, and scaffold structures that mimic the physical and mechanical properties of real organs [1,34,36]. Biologically, selecting appropriate cell types—whether primary cells, immortalized cell lines, or stem cells—is of utmost importance to accurately replicate organ structure and function. Optimizing culturing conditions, including temperature, pH, and nutrient supply, is essential for preserving cell viability and functionality. Integrating these biological and technical elements is vital to creating an environment that reflects the physiological conditions of the organism. The real-time control of culture parameters increases the accuracy and quality of results. Therefore, the effectiveness of OOC models depends on combining technical designs with biological factors, thereby facilitating a deeper understanding of physiological processes and disease mechanisms in both humans and animals (Figure 2).

### 2.1. Technical Aspects of the OOC Models’ Design

The development of OOC models requires careful consideration of the biomaterials employed in the fabrication processes. These biomaterials must meet specific criteria to ensure the most accurate reproduction of the physiological conditions in the body’s organs and tissues. The material used for the microchip must be biocompatible, supporting cell adhesion and function without exerting adverse effects [37]. Additionally, the biodegradability of the microchip material can help with tissue remodeling, which is essential for long-term applications [38]. The mechanical properties of the material should allow for the replication of the target organ’s response to various physicochemical parameters of the medium, thereby ensuring an accurate simulation of physiological conditions [14]. The resistance of the microchip material to sterilization and purification processes is also essential, as maintaining a sterile microenvironment within the microchip is necessary for optimal cell growth and viability [18,39]. Additionally, the origin of the material used in microchip construction significantly impacts the reliable performance of OOC models. A consistent composition of the material ensures uniform results. When using natural polymers, such as collagen or hyaluronic acid, it is often difficult to retain consistent conditions of the system due to their biological variability, which can be influenced by factors such as the age and health of the source organism. In contrast, synthetic polymers like PDMS offer a greater control over material properties and production processes, resulting in more consistent batches. This makes synthetic polymers more suitable for applications requiring high reproducibility and precision, such as drug testing and disease modeling [37]. Another critical technical aspect is the surface treatment and patterning of OOC models. This is essential for creating biologically relevant environments that mimic the complex 3D structures of tissues and organs. These techniques enhance cell attachment, growth, and differentiation by modifying biomaterial surfaces, promoting improved cell adhesion and organization. As a result, more accurate tissue modeling is achieved, increasing the effectiveness of OOC applications in tissue engineering, drug testing, and disease modeling [14,39]. Finally, the material must exhibit optimal rheological properties to replicate the physiological conditions of tissues and organs. These properties, including viscosity and shear stress, significantly influence fluid dynamics and cellular behaviors like adhesion, proliferation, and differentiation in the OOC model. By accurately mimicking the mechanical environment of specific tissues, rheological properties help create more realistic and functional tissue models, thereby increasing the reliability of OOC systems in biomedical research and therapeutic applications [40]. These factors illustrate the necessity for careful material selection to develop consistent and accurate OOC models, which are essential in biomedical research. Improving the materials and methods employed enables the development of more effective tools for studying biological processes and testing new treatments.

Currently, the rapid development of OOC technology is being observed. Various materials exhibit unique properties that make them suitable for the design of specific microchip components (Table 1). The traditional use of PDMS is declining due to its limitations, such as the absorption of small molecules, which can lead to erroneous results in drug studies [41]. Silicon, well-known for its role in electronics, can be utilized to create inexpensive, disposable devices. Silicon glass microfluidic devices are commonly used in high-throughput applications, such as flow-through microreactors for hazardous reactions or the synthesis of toxic chemicals and nanomaterials [42]. However, their use in OOC systems is less common due to their higher fabrication cost and inferior biocompatibility compared to PDMS [43]. Thermoplastics, such as polycarbonate and cyclic olefin copolymer, have gained popularity due to their ease of manufacture and availability in different thickness and porosity [44,45]. These materials are often used as microporous membranes to support cells in OOC models [41]. Epoxy resins and adhesives are occasionally employed in glass or silicon devices. However, there is still an ongoing search for elastomers that do not absorb molecules while remaining flexible, simple to use, and easy to fabricate [46,47,48]. Combining thermoplastics with hydrogels or elastomers in composite materials has been shown to improve cell viability and more effectively replicate natural tissue environments [49,50,51]. Technological advances, such as 3D printing, are significantly improving the functionality of OOC models [52,53,54]. Another 3D printing method, direct laser writing, has also contributed to the advancement of OOC fabrication. This high-precision technology uses a focused laser to solidify liquid materials, enabling the creation of intricate structures as small as 100 nanometers by building them point by point and layer by layer [44]. Overall, the trend is shifting toward materials that overcome the limitations of PDMS, focusing on cost-effectiveness, ease of fabrication, biocompatibility, and high-throughput manufacturing to more accurately replicate organ physiology for various studies, including drug screening and toxicology testing.

In summary, the careful selection and use of innovative biomaterials are critical to the development of OOC technology. By eliminating the limitations of traditional materials and adopting new fabrication techniques, researchers can create more accurate and reliable OOC models. These innovations have the potential to markedly enhance drug discovery, toxicology research, and personalized medicine, ultimately leading to more effective treatments. Integrating these new approaches has the potential to revolutionize medical research and clinical applications.

### 2.2. Biological Aspects of OOC Model Design

The starting point in designing an OOC model is the selection of the organ or biological structure to be replicated. To achieve this, appropriate cells must be utilized. Ideally, all biological structures would originate from the primary cells of the selected organ. However, obtaining primary cells, especially for most human organs, is very legally and technically complicated. Nevertheless, some OOC models of the BBB are successfully created using primary human brain-derived microvascular endothelial cells. These cells form the endothelial lining of the BBB, which is crucial for regulating the passage of molecules and cells into and out of the central nervous system (CNS) [55]. Another primary cell type, human brain vascular pericytes, is also used in BBB OOC models. These cells support the structure and function of the BBB by interacting with endothelial cells and astrocytes. However, in most cases, OOC models utilize cell lines derived from primary cells [56]. The use of cell lines allows for a greater cell expansion during culturing, making them more practical for large-scale studies. However, primary cells more accurately represent the native phenotype of the organ’s cells compared to monoclonal cell lines. Improving the quality of cell line culturing can be achieved through techniques such as perfusion, co-culturing, or a combination of both [57].

Stem cells are another type of cells that can be utilized in OOC systems, as they can differentiate into various cell types, facilitating their use in disease modeling and drug toxicity testing [30]. Theoretically, stem cells are an ideal, self-renewing cell source for OOC applications. The most commonly used stem cells are adult stem cells, embryonic stem cells, and induced pluripotent stem cells (iPSCs). The predominant adult stem cells are mesenchymal stem cells, typically derived from adult tissues. They are multipotent, meaning their differentiation potential is limited to specific cell types, primarily from mesodermal lineages such as bone, muscle, fat, or cartilage [58]. This limitation makes them less advantageous than iPSCs, which are pluripotent and capable of differentiating into cell types from all three germ layers, offering greater flexibility for OOC models [59]. Human embryonic stem cells are derived from the blastocyst or inner cell mass of embryos. Although they possess totipotent or pluripotent properties, enabling differentiation into various human cell types, their application in personalized medicine for disease modeling and drug evaluation is limited by ethical concerns, and technical challenges. Despite these limitations, this type of cell has proven useful in OOC models. In studies on amyotrophic lateral sclerosis (ALS), neural stem cells derived from human embryonic stem cells were successfully introduced to form motor neuron spheroids. Similarly, neural progenitor cells derived from the human H1 embryonic stem cell line were used to recreate brain tissue in an OOC model of the brain–gut–microbiota axis in metabolomics research [60].

In neuroscience, a pure population of iPSC-derived dopaminergic neurons is of great significance for studying Parkinson’s disease, which primarily affects this type of neurons in the substantia nigra [61]. This cell type is suitable for use in OOC models designed to simulate various neurodegenerative diseases. One such example is the application of iPSC-derived skeletal muscle cells to study ALS pathology. These cells enable the recreation of mature 3D muscle fiber bundles that retain their structure and functionality over time, in contrast to the mouse C2C12 myoblast cell line [60]. The main advantage of iPSCs over embryonic stem cells is that they are derived from somatic tissues of adult individuals rather than from embryonic cells, mitigating potential ethical issues. Moreover, because iPSCs are sourced from individuals with known disease phenotypes, they provide a valuable tool for the development of personalized disease models and drug screening [62,63].

In summary, the selection of cell types ultimately depends on the structure of the organ being replicated. Among the most optimal options are stem cells, particularly iPSCs, which are favored due to their relatively straightforward procurement process and their ability to differentiate into nearly all cell types. This versatility renders them exceptionally useful in the development of OOC models. Applying iPSCs enhances the physiological relevance of these models and provides a robust platform for studying complex organ function and disease mechanisms in vitro.

## 3. OOC Models in Neurosciences

OOC models have become valuable tools in neurosciences, particularly for investigating the BBB, brain–gut–microbiota axis, and neurodegenerative diseases (Table 2). The latter pose a major public health threat, and incomplete understanding of their underlying mechanisms remains a major challenge for modern medicine [64]. OOC models can replicate organ structures, allowing for a detailed examination of disease mechanisms and testing novel treatments. This technology represents a promising tool for advancing the understanding and treatment of many prevalent conditions, including ALS and Parkinson’s disease.

### 3.1. Blood–-Brain Barrier-on-a-Chip

OOC technology is applied in various neurological studies, with the BBB representing a particularly prominent area of research. The most recent research is concentrated on several different aspects related to the BBB, but the most significant of these is the discovery of new drugs based on the ability to permeate this structure, as well as studies focusing on neuroinflammation and cancer metastasis [65,66]. The BBB is a selective physiological barrier that protects the brain from potentially harmful substances in the bloodstream, while permitting the entry of essential nutrients and the clearance of metabolic waste. It is primarily composed of endothelial cells connected by tight junctions, as well as astrocytes and pericytes [70]. Developing therapeutics targeting the CNS and advancing new drug research present significant challenges, particularly in translating these findings into clinical trials.

One of the major challenges in developing neurological drugs is the difficulty of penetrating the BBB [71,72,73]. Current research is focused on gaining a more comprehensive understanding of BBB mechanisms and developing new drug delivery technologies. BBB-on-a-chip aims to imitate a functional BBB by replicating 3D cellular spatial arrangements, intercellular communications, and organ-specific mechanical and biochemical gradients. An example of this application is a study conducted by Marino et al. [74], where a 3D model of a biomimetic and biohybrid BBB was developed. These authors fabricated microtubes as scaffolds for co-culturing endothelial-like bEnd.3 and U87 glioblastoma cell lines. Additionally, a mathematical model was created to assist in designing and characterizing the 3D microfluidic chip. Marino and colleagues employed fluorescent dextran to assess barrier permeability and transendothelial electrical resistance, thereby evaluating its functionality. The results demonstrated that the bio-hybrid BBB model successfully mimicked the barrier’s function, forming tight junctions and effectively preventing dextran diffusion. Furthermore, the model generated transendothelial electrical resistance values comparable to those observed in other 2D BBB models. Another study involving nanoparticle transport mechanisms within the BBB was conducted by Ahn et al. [75]. These authors aimed to develop a microengineered human BBB as an in vitro model for pre-screening drug candidates for neurological diseases. The model consisted of two compartmentalized layers designed to accommodate brain endothelial cells, pericytes, and a 3D astrocyte network. It successfully replicated key BBB characteristics, including cellular interactions, gene expression, low permeability, and the formation of a 3D astrocyte network with reduced reactive gliosis and polarized aquaporin-4 distribution. The model demonstrated distinct cellular uptake and penetration of the BBB through receptor-mediated transcytosis.

The NeuroVascular Unit offers a promising approach for studying BBB physiology and neurovascular interactions. This microfluidic model is designed to recreate the in vitro conditions of the BBB, incorporating vascular and brain chambers separated by a porous membrane to replicate the BBB microenvironment. Brown et al. [76] described the development of an OOC model capable of accurately replicating the physiological and structural characteristics of the BBB. The NeuroVascular Unit facilitated cell-to-cell communication and independent perfusion. To recreate the cellular composition of the BBB, the model included primary human brain-derived microvascular endothelial cells, pericytes, astrocytes, and neurons. The formation of tight junctions was assessed using immunocytochemistry and transendothelial electrical resistance measurements. In another study by Boghdeh et al. [55], a gravity-flow NeuroVascular Unit was used to recreate the multicellular environment of the BBB. The objective was to assess the therapeutic potential of omaveloxolone in maintaining BBB integrity and reducing inflammation. As in the previous model, this microfluidic BBB model also consisted of human brain-derived microvascular endothelial cells, astrocytes, and pericytes.

**Figure 3:**
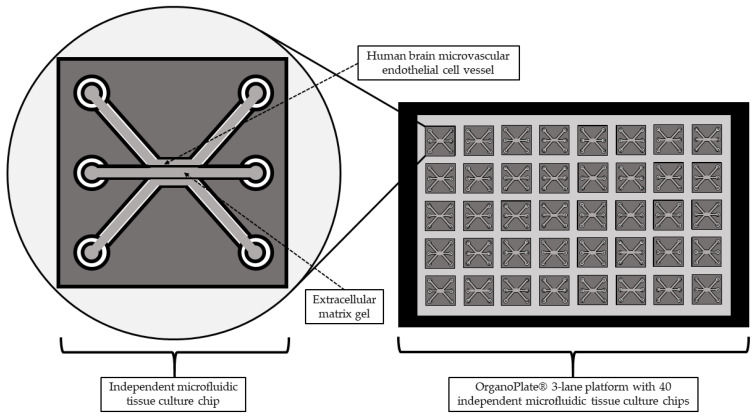
Schematic diagram of the blood–brain barrier-on-a-chip recreated on the OrganoPlate^®^ 3-lane 40 independent microfluidics tissue culture chips. The vessel formed from the human brain microvascular endothelial cells grows in the top lane against the extracellular matrix gel in the middle lane. Adapted from Nair et al. [25].

Nair et al. [25] constructed a model of human BBB-on-a-chip to study the effects of pro-inflammatory cytokines on monocyte migration to the BBB (Figure 3). Primary human brain microvascular endothelial cells (ACBRI 376, Cell Systems) were cultured in Promocell MV-2 medium and used between passages 5 and 10. T cells were isolated from peripheral blood mononuclear cells from a healthy donor, using density gradient centrifugation with Ficoll and magnetic bead separation. THP-1 monocytes were cultured in RPMI 1640 medium supplemented with 10% fetal bovine serum, 1% GlutaMAX™, and 1% penicillin–streptomycin. All cells were maintained at 37 °C with 5% CO_2_ and regularly tested for mycoplasma contamination, ensuring high-quality inputs for the model. The researchers utilized the OrganoPlate 3-lane culture platform, which provided a controlled experimental environment through its specific lane and phase guide dimensions. The lanes were prepared with a 4 mg/mL rat-tail collagen-I extracellular matrix, followed by Matrigel-GFR coating. After polymerization and coating, HBMECs were seeded at 10,000 cells/μL using a passive pumping method, and the plate was incubated to allow cell adhesion against the gel. The culture medium was regularly replenished, and the bottom lanes were either left empty or perfused with a special salt solution to promote optimal barrier formation. The OrganoFlow perfusion rocker was used to facilitate vessel formation and lumen perfusion, mimicking physiological conditions. Inflammation was induced in the model by apically exposing human brain microvascular endothelial cell tubes to varying concentrations of TNFα alone or combined with IL-1β for 24 h. The study demonstrated that inflammation impairs BBB integrity, alters cellular morphology, and promotes monocyte and T-cell adhesion and migration.

Motallebnejad et al. [77] developed an in vitro BBB-on-a-chip model using human iPSCs to address the limitations of animal studies in drug screening, disease modeling, and BBB disruption research. In this study, the cells were cultured in the device to form a confluent monolayer, and viability was assessed by calcein-AM staining. Impedance spectroscopy was employed to measure the transendothelial electrical resistance across the monolayer, a key indicator of barrier integrity. The results showed that the cells formed a tight barrier, as confirmed by immunocytochemistry and fluorescein permeability assays. The addition of TGF β1, a cytokine elevated in the plasma of patients with various diseases, led to a reduction in BBB integrity and increased expression of astrocyte activity markers. This mimicked the BBB disruption observed in pathological conditions.

### 3.2. Brain–Gut–Microbiota Axis-on-a-Chip

The brain–gut–microbiota axis is believed to play an essential functional role in the body. This bidirectional communication system connects the gut and its microbiota with the CNS through neural, endocrine, and immunological mechanisms. Recent research has suggested that the gut microbiota—comprising bacteria, fungi, viruses, and protozoa—might be one of the most critical factors influencing CNS function. The dysregulation of this axis has been implicated in various pathophysiological conditions, ranging from inflammatory bowel disease to neurodegenerative disorders. Manipulating the microbiome has been shown to affect neuronal activity in the CNS either via local interactions with enteric nerves that project to the brain or through the systemic circulation affecting organs, tissues, and immune cell interactions [78,79,80]. The modulation of the activity of regulatory processes at the CNS level by the microbiome can occur through bacterial metabolites such as short-chain fatty acids, secondary bile acids, or tryptophan metabolites. These molecules interact with enteroendocrine cells to modulate the secretion of gastrointestinal hormones, including ghrelin and obestatin. These molecules not only regulate appetite at the CNS level but also affect the gene and protein expressions of essential hormones of the gonadotropic and somatotropic axes [81,82].

In recent years, the disruption of the normal functioning of the gut–brain axis has been increasingly recognized as a contributor to the development of neurodegenerative diseases. Consequently, efforts have been made to recreate the brain–gut–liver axis using OOC technology. A ‘human physiomimetic model’ has been developed to more accurately represent the pathophysiology of human diseases. This model consists of several independent yet interconnected microphysiological systems (MPSs). When linked to an integrated pumping system and exposed to media perfusion, these in vitro models mimic specific organ functions. The goal of physiomimetic models is to reproduce key elements of complex disease states involving multiple systems, while maintaining a simplified experimental configuration of MPS for further research [83]. In Parkinson’s disease research, an experimental platform was designed, consisting of three MPS representing the intestine, liver, and brain, and assembled into a functional system through continuous medium flow. The gut MPS were established using primary colon epithelial cells cultured as organoids and loaded as single cells to form a differentiated primary epithelial monolayer. Myeloid cells were added to the basolateral side of the membrane to represent intestinal immune defense and modulation of epithelial homeostasis. The liver MPS comprised a co-culture of primary human hepatocytes and Kupffer cells, loaded onto a microperfused ‘liver scaffold’ for optimal oxygenation and nutrient flow. The brain MPS was composed of neurons, astrocytes, and microglia derived from human iPSCs, similar to previous BBB-on-chip models. This integrated MPS model provided valuable insights into the complex gut–liver–brain interactions, illustrating how these relationships influence the pathophysiology of Parkinson’s disease [83].

**Figure 4:**
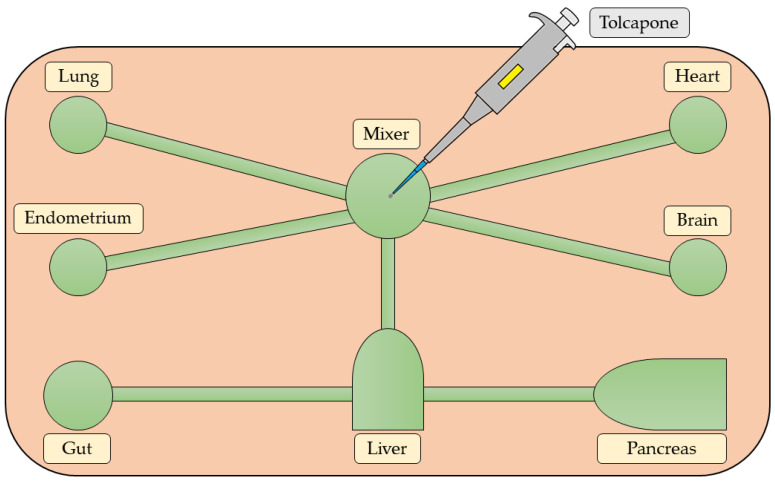
Schematic diagram of the “human-on-a-chip” system simulated on the microfluidic chip for the comprehensive study of tolcapone metabolite profiling and metabolomics. The microchip comprises seven interacting microphysiological systems (green): brain, pancreas, liver, lung, heart, gut, and endometrium, with a mixer chamber for systemic circulation and tolcapone dosing. The illustration was created using information from the article by Wang et al. [84] as a reference.

The brain–gut axis can also be used to study drug metabolism. Tolcapone, a Parkinson’s disease treatment, was applied in this model to investigate its metabolic pathway and the effects on endogenous metabolites and pathways in the human CNS. Wang et al. [84] introduced a multi-organ OOC model of ‘human-on-a-chip’ by interconnecting seven OOCs: brain, pancreas, liver, lung, heart, gut, and endometrium (Figure 4). Tolcapone was dosed and recirculated throughout the entire system via a mixer chamber. The study identified three new tolcapone metabolites and revealed significant alterations in 18 key biomarkers within the brain OOC. These alterations were primarily associated with disruptions in the metabolism of tryptophan, phenylalanine, glycerophospholipid, and aspartate. This research demonstrated the potential of the ‘human-on-a-chip’ model to emulate human drug responses in vivo, improving both drug efficacy and toxicity testing.

### 3.3. Amyotrophic Lateral Sclerosis-on-a-Chip

In recent years, many models have been developed to study the etiology, intracellular mechanisms, and potential treatments for ALS, a progressive neurodegenerative disease characterized by the degeneration of motor neurons, which eventually affects essential functions such as swallowing, speaking, and breathing [85]. Although no cure currently exists for ALS, the combination of tissue engineering, stem cell therapy, and novel OOC technology holds promise for advancing ALS research [86,87].

OOC models are increasingly being used to screen for substances with potential therapeutic effects in treating ALS-induced pathological changes. Cytoplasmic mislocalization of transactive response DNA-binding protein-43 (TDP-43) and fused in sarcoma (FUS) aggregates have been identified as ALS biomarkers, which are convenient indicators for studying the disease in motor neuron cultures [85]. OOC technology has been used to investigate the initiation and progression of TDP-43 aggregates, which accumulate in ubiquitinated inclusions in motor neurons affected by ALS. In a study by Chennampally et al. [88], a microfluidic model was developed to evaluate the therapeutic potential of rapamycin in ALS. This model used a linear concentration gradient of rapamycin in a 3D cell culture suspended in a gel matrix. The microchip chamber was loaded with neuromuscular cells derived from embryonic stem cells of transgenic mice expressing the ALS phenotype. Specifically, mutant embryonic stem cells derived from mice with TDP-43-A315T mutations were used in a model. These cells expressed Hb9::GFP under the Hb9 promoter, allowing motor neurons to be identified by green fluorescence. The mutant cells were isogenic, cultured from a C57BL/6 J background, and differentiated into motor neurons differentiation media containing Advanced DMEM/F12, Neurobasal media, and specific growth factors (CNTF, GDNF, BDNF, and NT-3), along with purmorphamine and retinoic acid supplements. The microdevice was fabricated using silicon wafers micromachined with photolithography and deep reactive ion etching to create microfluidic channels and a cell culture chamber. The silicon chip was bonded to a glass substrate to seal the channels, and small vias were introduced to allow for the diffusion of mediators into the gel-filled culture chamber. Optimized using the COMSOL software, the design allowed precise control of fluid flow and concentration gradients, mimicking tissue-like environments. The cell-laden gel matrix was prepared using Geltrex at 4 °C, dispensed into the microfluidic device at a density of 10^6^–10^7^ cells/mL, and set at 37 °C. Fluidic channels perfused the cells continuously with pre-equilibrated motor neuron differentiation media or rapamycin, maintaining consistent experimental conditions. The concentration gradient was validated using fluorescein diffusion, ensuring precise chemical control within the microchip. A traditional source-to-sink diffusion method was used to maintain a constant gradient of rapamycin. High, constant concentrations of rapamycin were introduced on one side of the microdevice chamber, while a lower concentration diffused freely on the opposite side of the chamber. Specifically, the rapamycin gradient was established by introducing 2 μM rapamycin into one channel and 0 μM rapamycin into the opposite channel, resulting in a linear concentration gradient from 2 μM to 0 μM across the chamber. The flow rate was maintained at ~100 μL/h to ensure consistent exposure conditions for the motor neuron cultures over seven days. This spatial gradient was used to assess the viability of ALS cells at different locations in the chamber. The microfluidic model developed in this study proved effective in evaluating rapamycin’s therapeutic potential in treating ALS-related motor neuron degeneration. Its design allowed for high-throughput drug screening, real-time monitoring, and customizable experimental conditions, thereby demonstrating its value as a versatile platform for ALS drug discovery and screening.

A more complex model for studying ALS-induced changes has been developed by Osaki et al. [60]. This group recently designed a motor unit model with neuromuscular connections using a co-culture of motor neurons and skeletal muscle cells, creating an ALS-on-the-chip simulation. The model is invaluable for studying the pathophysiological interactions between motor neurons and skeletal muscle cells, enabling the real-time observation of motor neuron cell death, synapse formation, neurodegeneration, or muscle cell contraction, and atrophy. The model is based on three different tissues: 3D skeletal muscle bundles, iPSC-derived motor neuron spheroids from a patient with sporadic ALS, and light-sensitive channelrhodopsin-2-induced motor neurons. These tissues were placed and cultured on separate microchips but connected to a single microfluidic system. During culture, motor neuron axons gradually grew and formed neuromuscular connections with muscle fiber bundles. This ALS-on-the-chip model was used to assess the therapeutic effect of concurrent administration of rapamycin and bosutinib. The integration of optogenetics technology into this ALS model may contribute to understanding ALS pathogenesis and high-throughput drug screening, facilitating the identification of potential treatments for the disease.

### 3.4. Parkinson’s Disease-on-a-Chip

Parkinson’s disease is a common neurodegenerative disorder affecting over 6 million people worldwide and emerging as a leading cause of neurological disability [89]. It is characterized by the presence of Lewy bodies and Lewy neurites, primarily composed of aggregated α-synuclein, leading to neuronal degeneration in the substantia nigra and other brain regions. The pathology of Parkinson’s disease is characterized by depigmentation and neuronal death in the substantia nigra and locus coeruleus, driven by mechanisms such as apoptosis, autophagy, mitochondrial dysfunction, and oxidative stress. Despite extensive research, the precise mechanisms of the disease and the role of Lewy bodies in its progression remain elusive [90].

A study by Fernandes et al. [91] introduced a microfluidic cell co-culture model designed to investigate the molecular mechanisms of Parkinson’s disease and other synucleinopathies. This model consisted of two PDMS layers containing two cell culture chambers connected by three channels. The system included channel inlets for introducing cells and media, outlets for each chamber, allowing for controlled cell–cell communication and real-time monitoring, as well as integrated pneumatic valves for fluid flow control. The model used H4 neuroglioma cells, transfected with an α-synuclein-green fluorescent protein to study protein spreading, and N9 microglial cells, activated with lipopolysaccharide to induce inflammatory responses. This model allowed for controlled communication between the two cell populations, simulating paracrine signaling and enabling the study of α-synuclein spreading and neuroinflammation. The results indicated the usefulness of the model in investigating the release and spread of α-synuclein, as well as the effects of microglia activation in neuron-like cells.

Similarly, De Rus Jacquet et al. [92] developed a microfluidic brain-chip model to investigate the effects of mutations characteristic of Parkinson’s disease on astrocytes, vascular changes, and BBB impairments. The microchip mimicked the NeuroVascular Unit model with a three-lane system that co-cultured brain microvascular endothelial cells, pericytes, and astrocytes in separate compartments. Induced pluripotent stem cells with Parkinson’s disease-related mutations from female donors were used to obtain astrocytes. The top lane of the model served as the vascular compartment, where brain microvascular endothelial cells formed an endothelial-like vessel, while the bottom lane acted as the brain compartment, housing pericytes and astrocytes. The brain-chip model was treated with a small-molecule inhibitor of the MEK1/2 pathway and LRRK2 mutation, both characteristic of Parkinson’s disease pathology, to modulate inflammation and angiogenesis. Astrocytes derived from iPSCs displayed mutation-induced changes in gene regulation, impairing barrier permeability, dysfunctional signaling, and altered cytokine levels. Treatment with the MEK1/2 pathway inhibitor significantly reduced pro-inflammatory cytokine secretion by the mutant astrocytes, restoring barrier integrity and reducing permeability. Conversely, the LRRK2 mutation inhibitor did not improve barrier integrity. These findings highlight the potential of MEK1/2 as a therapeutic target for Parkinson’s disease, as demonstrated in the developed microfluidic brain tissue microchip model.

## 4. The Introduction of the OOC Technology in Experimental Neuroendocrinology

Neuroendocrinology is an interdisciplinary field that examines the anatomical, biochemical, and functional relationships between the endocrine and nervous systems. Neuroendocrinology focuses on neurohormones and other biologically active substances produced in specific brain structures that regulate many functions under physiological conditions, in stressful situations, and during pathological changes within the CNS. Experimental neuroendocrinology and clinical neuroendocrinology can be distinguished as two branches of this field, both of which have seen significant advancements in knowledge. This progress is largely attributable to dynamic technological innovations that open new research opportunities.

As an example of how OOC technology can be applied, new microfluidic models have been developed to replicate various structures of the CNS. These models can generate dynamic spatial and temporal microenvironments similar to those occurring during vertebrate neuronal development. Preliminary experiments have shown that microfluidic devices not only support stem cell growth but also enable the creation of customized chemical environments within the chips, such as morphogen gradients, resulting in neural tube development similar to in vivo conditions [93]. In the other studies, 2D and 3D models were used to recreate the highly complex structure and function of the hypothalamic–pituitary (HP) axis [94,95]. However, the multicellular complexity, spatial environment, and complex neuroendocrine interactions within this axis make “functional replication” difficult. The HP axis is one of the most essential systems in the organism, and reciprocal neuroendocrine communication between the hypothalamus and pituitary plays a vital role in maintaining energy homeostasis and regulating growth and reproductive processes [96,97]. Therefore, the application of OOC technology may allow for effective restoration of the complex physiological processes of the HP axis [14].

**Figure 5:**
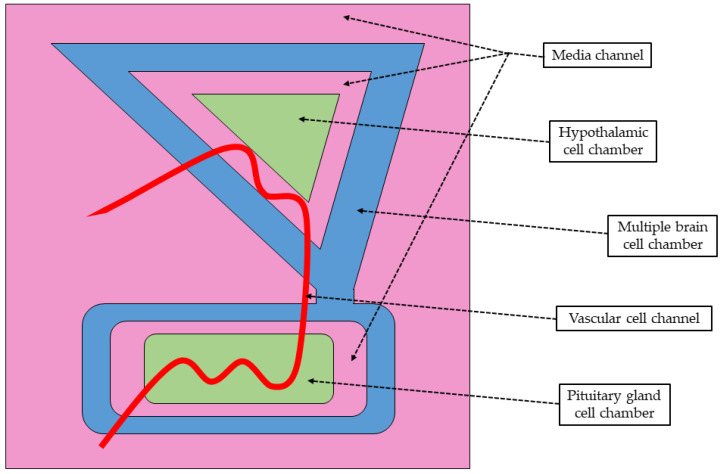
Schematic diagram of the hypothalamus–pituitary (HP) axis-on-a-chip developed to recapitulate the reciprocal neuroendocrine crosstalk between hypothalamus and pituitary gland. Vascular endothelial cell-coated media channels (red) interconnect the hypothalamus and pituitary gland chambers (green) to allow their bidirectional crosstalk within the HP axis-on-a-chip model. These two chambers, and connecting them vessel channel, are surrounded by an additional chamber loaded with multiple types of brain cells (blue) to simulate the natural multicellular microenvironment of the brain. Culture media chambers (light pink) provide conditions simulating the microenvironment of the hypothalamus and pituitary gland. The casting mold for the chip was fabricated with PLA-based 3D printing. The illustration was created using information from the article by Park et al. [54] as a reference.

Park et al. [54] employed existing anatomical knowledge and computer-aided design to create an ‘HP axis-on-a-chip’ mold, which was subsequently printed using 3D technology (Figure 5). The mold, formed using PDMS, was designed with two primary compartments—the hypothalamic (7 mm diameter, 4 mm thickness) and pituitary chambers (13 mm diameter, 4 mm thickness)—connected by a vessel channel lined with vascular endothelial cells to enable bidirectional neuroendocrine communication. To enhance biocompatibility and structural stability, the chambers were structured with a blend of natural polymers, including Type I collagen, hyaluronic acid, fibrinogen, and thrombin. After solidifying the PDMS solution injected into this mold, an experimental model was recreated consisting of several chambers connected to form a functional chip. This model mimicked the multicellular properties of complex brain tissues to replicate the interactions of different cell types in the HP axis. It featured two compartments: the hypothalamic chamber and the pituitary chamber, loaded with commercially available mouse GT1-7 hypothalamic nerve cells, rat RC-4B/C pituitary cells, and human umbilical vein endothelial cells. Cells were cultured in optimized media (DMEM or EBM-2, supplemented with essential growth factors and 5% FBS) to support the viability and neuroendocrine functionality. These two microdevices were connected by medial channels lined with vascular endothelial cells to enable neuroendocrine communication. Additionally, to more accurately reflect the complex microenvironment of the HP axis and its interaction with different types of neuronal or non-neuronal cells, the outer space of both ventricles was populated with various cells naturally occurring within hypothalamic and pituitary structures. These included human neural progenitor cells, microglial cells, and astrocytes cultured in cell-specific media supplemented with FBS. Mixed with the natural polymer blend, they established a multicellular network replicating brain tissue interactions. The chip was maintained with a mixed culture medium refreshed every two days to ensure long-term viability.

**Figure 6:**
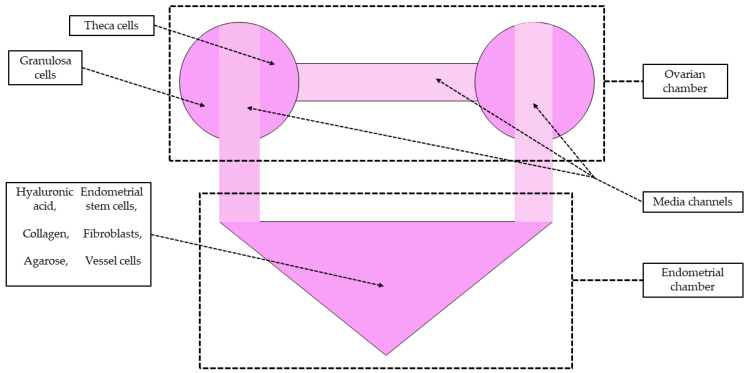
Schematic diagram of the microfluidic female reproductive system reflects the bidirectional endocrine cross-talk and complex multicellular structures by integrating various cellular components of the human uterine endometrium and the ovary with several biodegradable natural polymers. The microfluidic chip comprises ovarian and endometrial chambers (dark pink) interconnected via media channels (light pink). The illustration was created using information from the article by Park et al. [98] as a reference.

The same research group also developed a model of endometrial–ovarian OOC (E-O OOC) [98]. This microdevice was developed using various types of human endometrial and ovarian follicular cells (Figure 6). The cellular components of the endometrial chamber consisted of endometrial stem cells, stromal cells, and vascular endothelial cells, all isolated from uterine endometrial tissues. For the ovarian chambers, granulosa and theca cells were employed, which were extracted from ovarian follicular fluid samples obtained during in vitro fertilization procedures. As in the HP axis OOC, this model also utilized computer-aided design and 3D printing to create PDMS-based chips to imitate the anatomical structure of the endometrium and ovary. The main advantage of this model, similar to the HP OOC axis described above, is its ability to facilitate bidirectional hormonal communication between the endometrium and the ovaries. The results demonstrated that E-O OOC provided high cell viability, optimal hormone secretion, and receptor expression, underscoring its reliability as a tool for studying reproductive physiology. It should be emphasized that both models—HP axis OOC and E-O OOC—have been developed not only for physiological studies but also for evaluating the efficacy and toxicity of potential drug candidates.

## 5. Benefits and Limitations of the OOC Technology

**Figure 7:**
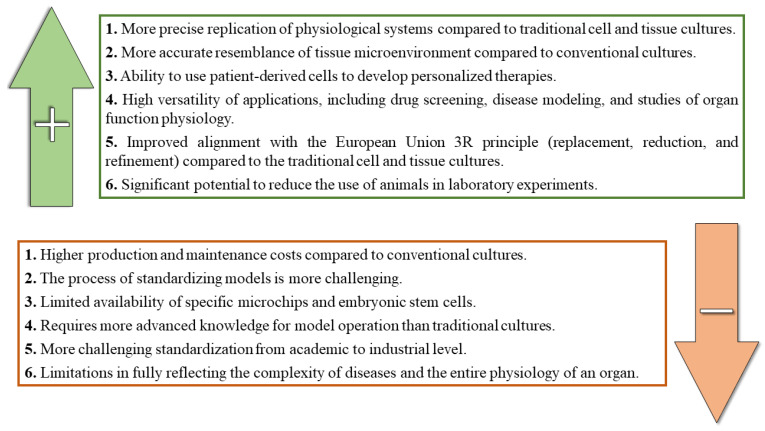
Advantages and disadvantages of OOC technology.

OOC technology presents a promising alternative to traditional cell and tissue culture methods commonly used in basic and applied research. Currently, scientific evidence shows numerous successes in replicating individual organs, tissue systems, and even entire organ systems using OOC models [99,100,101]. However, it is crucial to acknowledge the existing limitations of OOC technology (Figure 7). Despite these challenges, the experimental models created using this technology have become invaluable sources of knowledge and tools for the effective use of OOC in future research.

Microenvironments reproducible in OOC models derived from multiple cell line types offer a substantial advantage over traditional mono- or co-cultures by better mimicking tissue and multicellular structure. In addition, the composition of the chip components frequently reflects the anatomical architecture of the target structures [52]. Another advantage of OOC is the precise control it offers over the internal microchip environment, reducing variability between measurements [102]. These models are particularly valuable for drug screening in disease modeling and identifying potential toxicity markers. Furthermore, the ability to simulate entire disease entities within a single microdevice, due to the complex design of OOC chips, facilitates a more precise understanding of disease etiology, enabling the development of new drugs and therapies. Importantly, OOC also contributes to reducing the use of animals in experiments, offering substantial ethical and practical benefits.

As with any emerging technology, OOCs have certain limitations. While the aforementioned examples demonstrate the utility of OOCs in modeling neurodegenerative diseases and replicating biological systems, it is essential to recognize that these models remain simplified versions of living organisms. Translating results to in vivo conditions presents a significant challenge to bioengineering. Moreover, developing intricate microchip architectures requires specialized expertise in microfabrication, cell culture, and microfluidics, posing a barrier to the broader adoption and implementation of OOC technology.

Another significant issue is standardization. Current OOC models operate at a microscale and translating them into commercially viable solutions presents further difficulties. Implementing these solutions on an industrial scale requires developing dedicated approaches and creating novel solutions. One of the most notable drawbacks of current organ-on-a-chip technology is its cost. Microchip manufacturing demands a high degree of precision to ensure the reliable reproduction of complex tissue architecture and to provide optimal conditions for cell culture. An OOC model is not merely a microchip, but also an array of microdevices for measuring and controlling culture conditions. The financial burden of equipping an OOC model with the appropriate devices raises questions about the feasibility of developing various solutions on an industrial scale.

As mentioned above, OOC technology is not the only alternative to in vitro models used in experimental research. Particularly in drug development and safety, organoids, spheroids, and other three-dimensional tissue cultures offer some advantages over traditional two-dimensional cultures [103]. Most organoids contain only a fraction of the cells present in the actual organ, and the reconstitution of functional, stable tissues requires methods that allow the introduction of other cellular systems, such as the cardiovascular system. As a result, 3D culture models are limited in their ability to reproduce complex physiological functions at the organ level and clinically relevant disease manifestations that involve multiple tissue types. In addition, OOC offers the ability to replicate the microenvironment of a tissue or organ by regulating the movement of fluids (e.g., the test drug medium) and enabling the simulation of a variety of mechanical stimuli [104]. This unique feature of OOC systems enables more comprehensive and accurate replication and prediction of complex drug responses in living organisms. In addition, OOC models allow the real-time visualization and quantitative analysis of the biological processes taking place, which is often not possible with organoids and spheroids [105]. With the appropriate use of OOC models, they can be integrated to form a model of a multi-organ system. This, in turn, allows the systemic interactions between multiple organs to be ‘captured’ to more accurately determine the pathogenesis of multi-organ diseases [106]. The increasing use of OOC in biomedical research shows that it can outperform conventional models, thereby accelerating drug discovery and personalized medicine. As a result, OOC technology is increasingly recognized as the new standard for modeling human-relevant conditions in biomedical research.

In summary, the benefits underline the essential need for continued advancement of OOC technology, which would ultimately benefit researchers, manufacturers, healthcare workers, and patients alike. Despite some certain disadvantages and challenges, the rapidly evolving OOC technology is expected to mitigate these limitations over time.

## 6. Future Direction of the OOC Technology in Neurosciences

The development of OOC technology requires the use of the latest technological achievements and the generation of more sophisticated approaches. Artificial intelligence (AI) has emerged as a promising solution for accelerating development and shaping multiple technological domains. An important area of AI development is the creation of models utilizing machine learning techniques, particularly deep learning [107].

Several key applications involving AI algorithms in OOC models have emerged in recent years [108]. Deep learning-based networks can stabilize flow conditions in microfluidic platforms by automatically adjusting parameters to eliminate performance inconsistencies, particularly improving vascularization in OOC models [109]. Additionally, machine learning algorithms, such as multiple linear regression and random forest, can assess the oxygen transport capacity and other morphological indicators of vascular networks within OOC systems, thereby aiding in evaluating their biological functions [110]. Moreover, the integration of spectroscopy, multisensor and microscopy systems with machine learning for data-driven optimization helps to regulate microenvironmental parameters and ensure accurate organ function and drug response in OOC models—an essential aspect of drug screening [111]. In the lung OOC model, deep learning facilitates the real-time monitoring of mechanical stretch, optimizing tissue mechanical forces and detecting cellular processes while preserving cell viability [107]. These applications illustrate the growing role of AI in enhancing the functionality and reliability of OOC models. Integrating AI algorithms into OOC technology represents a promising frontier in biomedical research. Although current applications are limited, they demonstrate significant potential for improving the precision and functionality of OOC models. Future advancements will likely require extensive research and interdisciplinary collaboration to fully achieve the benefits of combining OOC technology with AI. This synergy has the potential to revolutionize drug testing and other areas in biomedicine, paving the way for more accurate and efficient research methodologies.

Another promising direction for the future development of OOC technology is personalized medicine. For OOC models to accurately reflect the physiology of human organs, it is essential to select appropriate biological resources. Immortalized human cell lines are widely used for research due to their ease of culture and rapid proliferation. Nevertheless, they are afflicted by genetic variations, lack of cellular complexity, and inconsistent phenotypes, which limit their ability to accurately mimic human physiology. Primary human cells, while more representative, face challenges such as limited proliferation, donor variability, and ethical concerns, which restrict their scalability and consistency in research. To overcome these limitations, there is a growing focus on using human stem cells, which have the potential to create more accurate and personalized tissue models. These stem cells can better replicate the complexity of human tissues and improve the predictive power of drug development research and applications in OOC technology [112]. This development will allow for a more accurate replication of tissue models and personalized medicine, positioning it as a key focus for future OOC development. OOC models offer a significant advantage in studying rare diseases in the field of personalized medicine. They facilitate the long-term, real-time monitoring of physiological processes, providing critical data unavailable in conventional laboratory experiments or animal models. Using machine learning, data can be analyzed in real time to gain insights into disease progression at the molecular level and identify its underlying mechanisms [107]. By incorporating iPSCs and patient-derived organoids, OOCs can provide models tailored to individual patients, allowing for highly customized personalized medicine that addresses the unique needs of patients with rare diseases [113,114].

The future evolution of OOC technology will undoubtedly aim to improve the quality of the produced microdevices while increasing the efficiency and throughput of data processing and analysis. AI will play a key role in advancing this field, utilizing machine learning models trained on large datasets to enable more the accurate, rapid, and comprehensive evaluation of results. This advancement has the potential to significantly reduce the time required to establish diagnoses and initiate early stage treatment, which is particularly important in certain diseases that lead to rapid and high mortality.

## 7. Conclusions

OOC technology is an invaluable asset in biomedical research, enabling precise disease modeling and developing and testing the efficacy of potential drugs. Moreover, this technology offers significant advantages for basic research, providing a more comprehensive understanding of the processes that regulate the functioning of individual organs or entire systems that modulate bodily functions. Its significant advantages over traditional cell and tissue cultures, combined with the integration of state-of-the-art advances in material engineering and microfluidics, render it an almost ideal model for addressing present scientific challenges. This versatile technology may be essential in neurology and neuroendocrinology due to its superior capability to replicate the complex neuronal systems in the brain and the entire nervous system.

Despite numerous reports demonstrating the prospective applications of OOC technology in diagnosing and developing therapies for neurodegenerative diseases, it remains a relatively novel approach. Most existing OOC models are mainly utilized in scientific research, with industrial standardization limited by the high costs associated with the sophisticated equipment required for individual models. Additionally, the requirement for extensive expertise in microchip design, microfluidics, and the creation of complete experimental systems, as well as maintaining cell and tissue cultures, significantly complicate the implementation of OOC as a new standard in biomedical research.

Nevertheless, OOC technology exemplifies the implementation of the European Union 3R principle (replacement, reduction, and refinement) concerning animal welfare, significantly reducing the number of animals needed for experimentation and potentially serving as a complete alternative to traditional animal models. In summary, the intensive development of microfluidic OOC technology promises significant benefits for human and animal health. It will act as a critical catalyst in advancing knowledge in neurobiology and across the broad field of medicine and related sciences.

## Figures and Tables

**Table 1:** Materials with potential OOC microchip applications.

Material	Advantages	Disadvantages	References
Poly(dimethylsiloxane) (PDMS)	Flexibility and cost-effectiveness, ease of fabrication, biocompatibility	Absorption of small molecules, leakage issues	[41]
Silicon glass	Precision and robustness, optical clarity, chemical compatibility	High fabrication cost, limited reusability, complex fabrication process	[42]
Thermoplastics	High optical transparency, solvent resistance, low gas permeability	Relatively high rigidity, complex manufacturing process, limited compatibility with certain photomaterials	[44,45]
Epoxy resins and adhesives	Advantageous mechanical properties, ability to integrate multiple sensors and actuators, hydrophilicity	Challenges with turning it hydrophobic, leakage issues	[46,47,48]
Thermoplastics combined with hydrogels or elastomers	Hydrogels: soft mechanical properties closely mimic native tissue, biocompatible microenvironment provides improves cell viability Polyurethane elastomers: optical transparency and biocompatibility, strong bonding capabilities	Hydrogels: swelling issues, challenging process of bonding with filters; Polyurethane elastomers: cytotoxic concerns, lower gas permeability than PDMS, processing challenges regarding degassing and molding	[49,50,51]

**Table 2:** OOC models in experimental science in recent years.

Organ/Structure Replicated	Disease/Condition Addressed	Cells/Tissues Used	Main Findings	Reference
Blood–brain barrier (BBB)	Drug discovery related to the BBB	Brain capillary-like endothelial cells (BCLECs) derived from human-induced pluripotent stem cells (hiPSCs)	A two-step differentiation protocol to derive BCLECs from hiPSCs, demonstrating that the combination of VEGF, Wnt3a, and retinoic acid significantly improved the expression of endothelial markers and barrier properties, including transendothelial electrical resistance and paracellular permeability. The derived BCLECs exhibited moderate expression of P-glycoprotein and responded to inflammatory stimuli, indicating their potential for modeling BBB function in vitro	[65]
BBB using the tissue chip platform “DigiTACK”	Neuroinflammation and related neurological disorders, with a focus on cytokine secretion dynamics	Primary mouse brain microvascular endothelial cells (mBMECs)	The DigiTACK platform enabled longitudinal monitoring of cytokine secretion from the mBMECs barrier, revealing significant differences in cytokine profiles between luminal and abluminal sides and demonstrating the potential for high-throughput analysis in studying CNS disease mechanisms	[66]
A gut–brain axis chip that mimics the intestinal and neural environment	Alzheimer’s disease model to evaluate the effects of gut microbiota-derived metabolites and exosomes	hiPSCs differentiated into induced neural stem cells and Caco-2 cells	Metabolites and exosomes derived from gut microbiota influenced neural growth, maturation, and synaptic plasticity, suggesting their potential as therapeutic candidates for neurodevelopmental and neurodegenerative disorders	[67]
A feto-maternal interface organ-on-chip (FMi-OOC) model	Ascending infections and their associated inflammatory responses that are significant risk factors for spontaneous preterm birth (PTB)	Primary human cells from the fetal membranes, including decidual cells, chorion trophoblasts, amnion mesenchymal cells, and epithelial cells	The FMi-OOC successfully demonstrated the propagation of lipopolysaccharide-induced inflammation from the maternal to fetal compartments, revealing distinct inflammatory cytokine profiles and highlighting the immune imbalance that can contribute to PTB. The model maintained key physiological characteristics of the in vivo environment, validating its utility for studying the feto-maternal interface	[68]
A six-chamber vagina–cervix–decidua-organ-on-a-chip (VCD-OOC) model that mimics the female reproductive tract during pregnancy	Ascending *Ureaplasma parvum* infection and its association with PTB	Vaginal epithelial cells, cervical epithelial and stromal cells, and decidual cells, all derived from immortalized human cell lines	*U. parvum* infection did not cause significant cell death or massive inflammation in the VCD-OOC model. However, combined with lipopolysaccharides, it induced a substantial inflammatory response. In vivo studies showed that the vaginal inoculation of *U. parvum* alone resulted in low PTB rates, while intra-amniotic injection significantly increased PTB rates	[69]
